# Nasal Glial Hamartoma: A New Type of Sinonasal Hamartomas

**DOI:** 10.7759/cureus.52781

**Published:** 2024-01-23

**Authors:** Nian Patel, Mehmet Server, Ahmed Bayoumi, Abdelrahman Ezzat Ibrahim

**Affiliations:** 1 Otolaryngology, United Lincolnshire Hospitals National Health Service (NHS) Trust, Lincoln, GBR

**Keywords:** seromucinous hamartoma, olfactory epithelial hamartoma, respiratory epithelial adenomatoid hamartoma, nasal chondromesenchymal hamartoma, inverted papilloma, nasal tumours, sinonasal hamartoma, glial hamartoma

## Abstract

Hamartomas are rare, tumour-forming, benign lesions that have been reported throughout the body that can resemble other malignant entities. Hamartoma subtypes can be distinguished based on their histological features. Sinonasal hamartomas may have presenting symptoms and radiological features that mimic other nasal neoplastic lesions. Therefore, it is essential to diagnose it accurately, as the treatment approaches can range from radical surgeries in malignant cases to a simple excision in hamartoma. In this paper, we report a novel case of sinonasal hamartoma, which demonstrates an unprecedented histological feature of glial tissue with astrocyte-like cells. Furthermore, we present the unconventional presenting symptoms and radiological features seen in this case that mimic the behaviours of nasal inverted papilloma (IP) lesions, thereby highlighting the need for careful investigation of such patients in order to distinguish both glial hamartoma and IP lesions. Concluding that identification of glial hamartoma as a new subtype of sinonasal hamartoma is crucial, as mistaking it for other lesions may subject patients to overly aggressive treatment and potential unnecessary harm.

## Introduction

Hamartomas are defined as a collection of cells or tissues expected at a given anatomical site but which are present in a pathological manner in amount, ratio, or distribution [1,2]. In the sinonasal region, hamartomas have been designated as epithelial, mesenchymal, and mixed types [3]. The previously reported types include nasal chondromesenchymal hamartoma (NCMH) [4], respiratory epithelial adenomatoid hamartoma (REAH) [5], seromucinous hamartoma (SMH) [2], and olfactory epithelial hamartoma (OEH) [6]. Although remarkable differences were noticed in their histopathological features, hamartomas still have close clinical and radiological significance. Therefore, histological investigations are required for a definitive diagnosis. Sinonasal hamartomas sometimes have radiological findings and present symptoms like the other nasal neoplastic lesions. Glial hamartoma, which is unfamiliar to see in such pathologies, might have specific characteristics that require further research and review. Considering the previous literature and its clinical and pathological characteristics, we will shed light on our case, figuring out its divergence.

## Case presentation

A healthy 30-year-old male presented with left nasal blockage with occasional nasal bleeding for two and a half years. He was working as a filmmaker, denied any recent history of hay fever or allergic rhinitis, and was a non-smoker. His general health was reportedly satisfactory, with no previous history of any nasal surgery. A flexible nasal endoscopic examination revealed multiple smooth, soft polypoidal lesions filling the left nasal cavity completely. The right nasal cavity was patent and free of any remarkable lesions. Our differential diagnoses included inverted papilloma (IP), allergic fungal sinusitis, hamartoma, and less likely inflammatory polyps.

**Figure 1:**
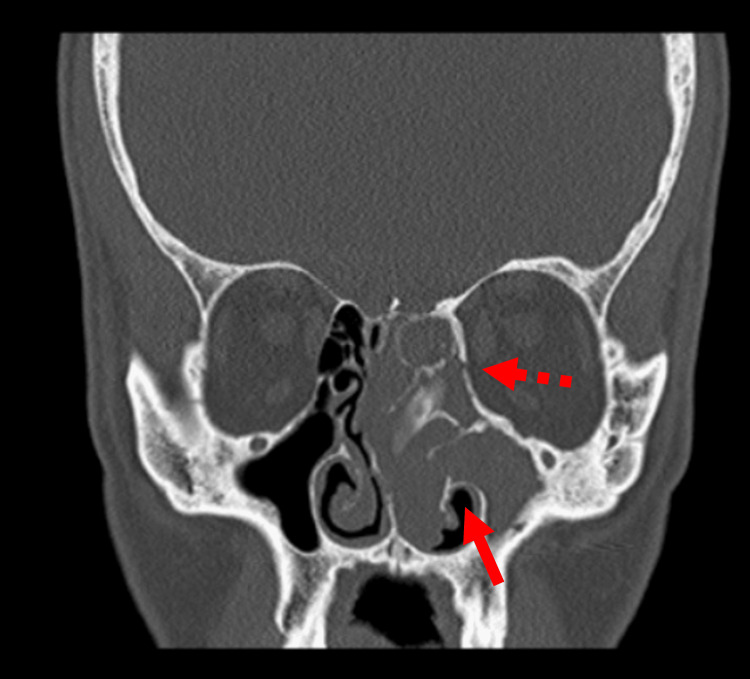
CT scan (bony window) coronal cut shows soft tissue lesion filling the left maxillary sinus extending into the nasal cavity with widening of the infundibulum. Focal hyperostosis at the anterior ethmoid air cells. The left ethmoid and left frontal sinuses also appeared filled with the mass. There was suspicious bony erosion, especially near the anterior medial orbital wall. Soft tissue lesion filling left maxillary sinus and nasal cavity (red arrow). Bony erosion near anterior medial orbital wall (red dashed arrow).

**Figure 2:**
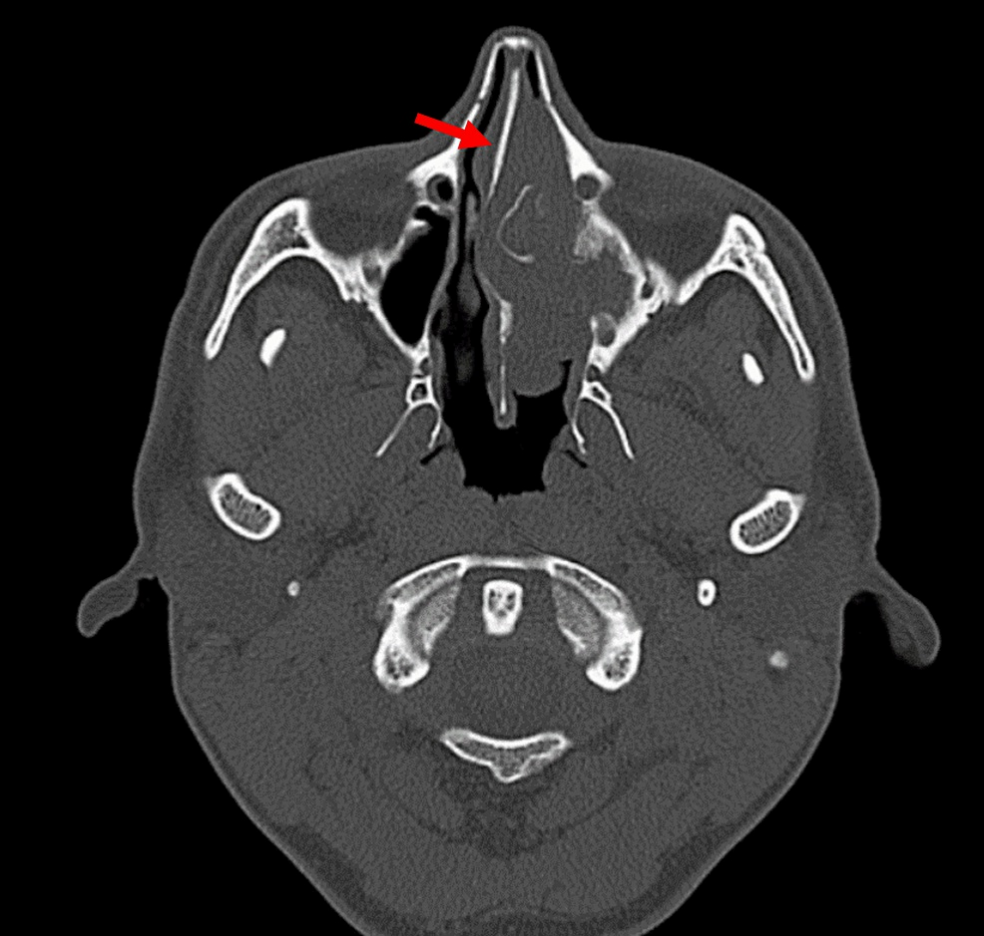
CT scan (bony window) axial cut shows soft tissue lesion filling the left nasal cavity completely with rightward deflection of the nasal septum. Rightward deflection of the nasal septum (red arrow).

**Figure 3:**
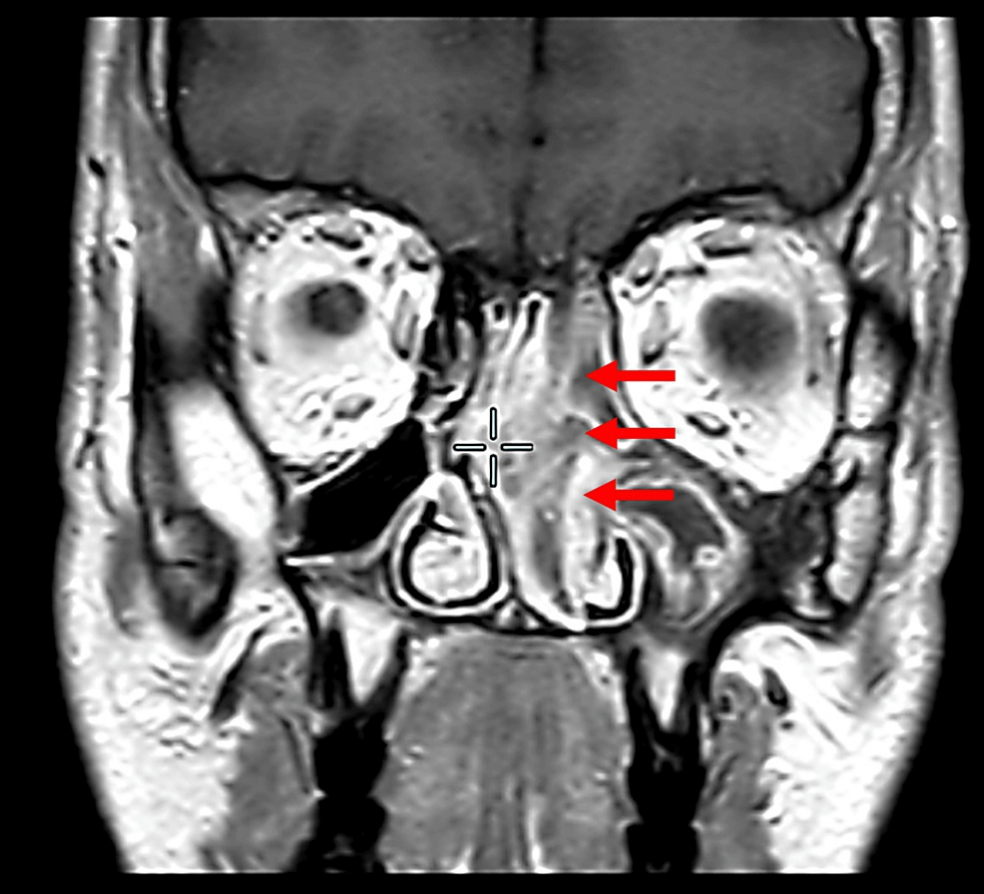
MRI scan (T2 weighted). There was a heterogeneously enhancing lesion noted in the left nasal cavity with a classic cribriform appearance extending into the left maxillary sinus causing ostial widening. The abnormal signal was also extending into the left ethmoid, sphenoid, and frontal sinuses. There was bulging of the medial wall of the left maxillary sinus into the nasal cavity with rightward deflection of the nasal septum. Cribriform appearance seen on the MRI scan (red arrows).

Initially, a CT scan of the paranasal sinuses was requested, which showed a left-sided soft tissue lesion occupying the left nasal cavity as well as the whole paranasal sinuses with bony erosion of the medial orbital wall. There was focal hyperostosis at the anterior ethmoid air cells (Figure 1). The nasal septum was significantly pushed towards the right side (Figure 2). Naturally, the patient underwent an MRI scan of the sinuses, which revealed the cribriform appearance, a significant sign of IP lesions (Figure 3).

**Figure 4:**
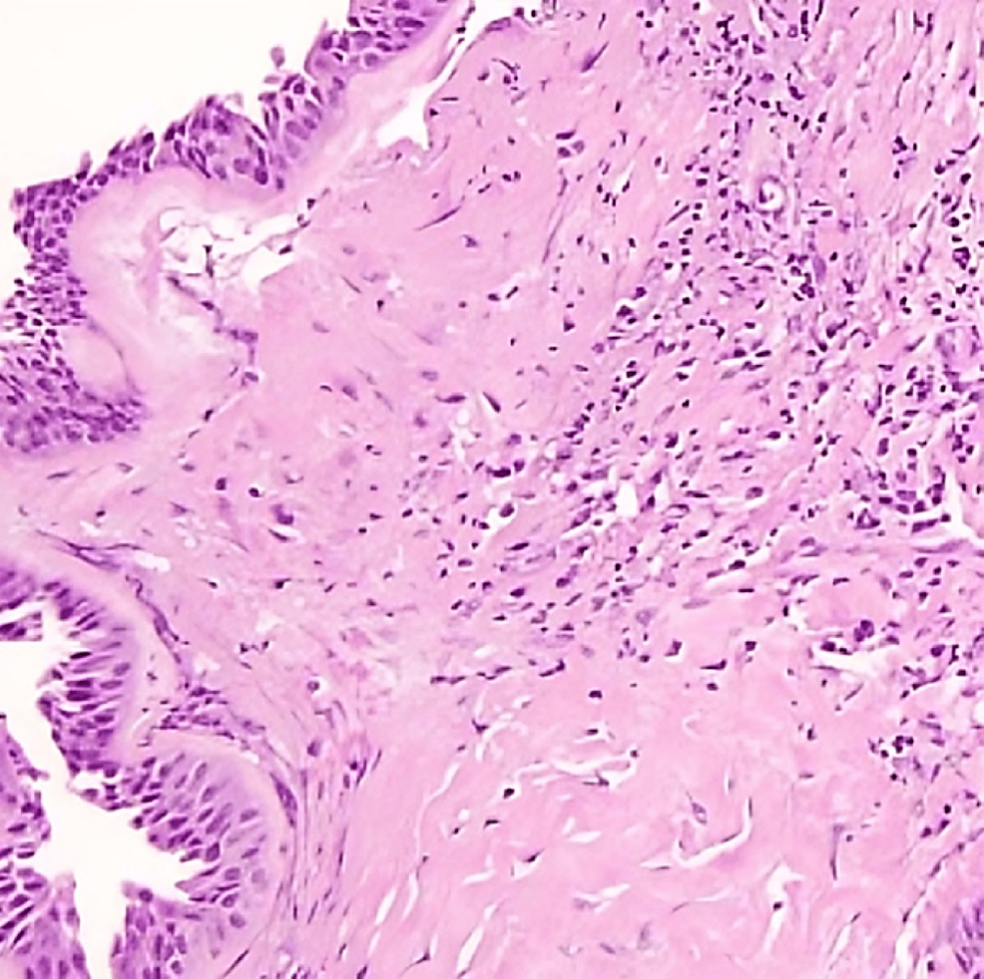
Histopathology shows inflamed mucosa with an abundance of seromucinous glands and thick-walled vessels. The structures were in a stroma consisting of glial tissue with astrocyte-like cells.

Subsequently, building up on the presenting symptoms and radiological findings, which were extremely suggestive of IP, the patient underwent left functional endoscopic sinus surgery and medial maxillectomy. The histopathological sample demonstrated multiple sections that showed similar features of inflamed mucosa (chronic inflammatory cells, including lymphocytes and macrophages) with an abundance of seromucinous glands and thick-walled vessels. The structures were in a stroma consisting of glial tissue with astrocyte-like cells (reactive gliosis) (Figure 4). Surprisingly, the histopathology report of the removed mass reveals features of nasal hamartoma, and no neoplasia or malignancy was seen.

## Discussion

The confusing clinical nuances of our case provoked our concern for the identification of glial hamartoma characteristics against other types of hamartomas as well as IP. Through reviewing the current literature, we analysed each type of hamartoma to get a wider picture of its features.

NCMH, REAH, and SMH are the most abundant subtypes documented in the literature. Only one paper narrated six patient stories of the fourth type (OEH) [6]. Nevertheless, it was meticulous and conclusive enough to describe this type accurately.

NCMH is a very rare, benign lesion of the sinonasal tract. Sixty-four cases are reported in English literature, of which most are infants and young children, often below the age of one [4]. To date, there have only been eleven cases of adult presentations of NCMH. Usually, adult patients presented with unilateral nasal obstruction, visible nasal mass, epistaxis, facial swelling, and pressure symptoms, whereas feeding difficulties since birth and respiratory distress syndrome are reported as presenting symptoms in infant cases. NCMH shows remarkable bony/cartilaginous erosion in most of the CT findings according to the variable sites within which they appear, including the nasal cavity, maxillary, ethmoid, sphenoid sinuses, and rarely the skull base. All reported cases were treated by endoscopic nasal surgery with no evidence of recurrence. Histopathologic features of NCMH include cartilage and focal mature bone covered in stratified squamous epithelium surrounded by reactive bony trabeculae [7].

REAHs are rare, benign glandular proliferations of the sinonasal cavity and nasopharynx, first described as a specific clinicopathological entity by Wenig and Heffner in 1995 [8]. REAHs in this region are often in the posterior nasal septum [9]. They typically arise in the third to ninth decades of life, with a male predominance (3:2) [10]. More than 70% of REAHs tend to be unilateral. Nasal obstruction, rhinorrhoea, epistaxis, recurrent chronic rhinosinusitis, facial pain, and hyposmia/anosmia are some of the symptoms of the disease [11]. It could be located bilaterally, with preference for the paranasal sinuses and olfactory cleft (OC). Radiographic evidence of OC expansion should raise the index of suspicion for REAH, although histological verification is required for a definitive diagnosis. Irrespective of clinical presentation, endoscopic removal appears to be curative [12]. Histopathologic features show cystic and glandular structures within a fibrous stroma lined by ciliated respiratory-type epithelium [13].

SMH, which can also be called epithelial hamartoma, glandular hamartoma, or microglandular adenosis, was first reported by Baillie and Batsakis in 1974 [14]. SMH is a benign glandular proliferation originating from the respiratory epithelium of the sinonasal tract and nasopharynx [15]. SMHs commonly develop in the posterior nasal cavity. Most patients are of middle or advanced age, with a male-to-female ratio of approximately 1:1.5 [15]. Common presenting symptoms include a long-standing history of nasal obstruction and/or epistaxis [14]. SMH should always be considered in the differential diagnosis of paediatric sinonasal disease, as one case was reported in such an age group [15]. SMH is less likely to have bony/cartilaginous erosion features in the CT scan findings. The treatment of choice for SMH is complete surgical excision by trans-nasal endoscopic approach [16]. Histologically covered by respiratory epithelium, it is comprised of lobular or haphazard proliferations of small to large glands and ducts that are lined by a single layer of cuboidal or flattened epithelial cells. The surrounding fibrous stroma is usually variable and often contains a lymphoplasmacytic inflammatory infiltrate [17].

Only six adult cases of OEH were reported in the literature [6]. They presented with recurrent sinusitis and unilateral nasal blockage. OEH are mainly located at the OC and are less likely to have bony/cartilaginous erosion features in the CT scan findings. Histologically, it resembles SMH but contains additional areas of olfactory epithelium in the surface epithelial lining and gland-like ductal invaginations [6].

IP is a rare sinonasal tumour that often occurs in adults during their 5th decade of life, with a mean age at diagnosis of 55 years of age [18]. Presentation is commonly delayed due to symptoms being non-specific, including nasal obstruction, anterior and/or posterior rhinorrhoea, headache, hyposmia or anosmia, epistaxis, or facial pain [18,19]. IP differs from other sinonasal tumours in three distinct ways: a relatively strong potential for local destruction, a high rate of recurrence, and a risk of carcinomatous evolution [18]. Microcalcifications within the lesion are found in about 20% of cases, often suggestive of an IP diagnosis [20]. Bone erosion is frequently seen, and CT images demonstrate features such as bone lysis that suggest malignancy [18]. There may also be evidence of focal hyperostosis at the implantation site of the IP [18]. Typical IP characteristics in MRI studies show an aspect of cribriform circumvolutions [18]. The case presented in this paper demonstrates similar radiological findings to those that have been reported in IP.

Histologically, IP demonstrates invagination of the superficial epithelium into the underlying stroma [18]. Treatment of IP is surgery by either endonasal endoscopic or external approach [18]. Patients diagnosed with IP are often subject to local recurrence; therefore, it is key that they are followed up carefully [18].

The key characteristics of each subtype of sinonasal hamartomas, including our novel glial hamartoma, are crucial to remember when assessing patients presenting with features suggestive of a unilateral mass. Especially when trying to exclude more sinister pathologies. Therefore, we have summarised these features in Table 1.

**Table 1:** Summary of sinonasal hamartoma subtypes and their characteristics SNH: sinonasal hamartoma; NCMH: nasal chondromesenchymal hamartoma; REAH: respiratory epithelial adenomatoid hamartoma; SMH: seromucinous hamartoma; OEH: olfactory epithelial hamartoma; GH: glial hamartoma; RDS: respiratory distress syndrome; OC: olfactory cleft

SNH types	Main age group	Presenting symptoms	Location	Bony/cartilaginous erosion	Significant histological cells
NCMH	Infants and young children	RDS/unilateral nasal obstruction	Variable	Present	Cartilage and focal mature bone covered in the stratified squamous epithelium surrounded by reactive bony trabeculae
REAH	3^rd^-9^th^ decade	Nasal obstruction and rhinorrhoea could be bilateral	Posterior nasal septum	OC expansion	Glandular structures within a fibrous stroma lined by respiratory epithelium
SMH	Middle or advanced Age	Unilateral nasal obstruction/epistaxis	Posterior nasal cavity/nasopharynx	Less likely	Lobular or haphazard proliferation of small to large glands and ducts that are lined by a single layer of cuboidal or flattened epithelial cells
OEH	Advanced age	Unilateral nasal obstruction, rhinorrhoea	OC	Less likely	SMH + areas of olfactory epithelium in the surface epithelial lining and gland-like ductal invaginations
GH	Young adult	Unilateral nasal obstruction/epistaxis	Sinuses	Present	Abundance of seromucinous glands and thick-walled vessels in a stroma consisting of glial tissue with astrocyte-like cells

## Conclusions

We believe that the existing subtypes of hamartomas, including our new one, shouldn’t be overlooked in our differential diagnosis of cases presenting with a unilateral nasal mass. Unfortunately, the initial investigations of sinonasal masses usually are not enough to confirm hamartoma as a benign lesion; thus, it is feasible for misdiagnosis, and patients could be treated overly aggressively. We would therefore advocate that patients presenting with features of a unilateral nasal mass be carefully investigated in order to avoid unnecessary and potentially harmful treatments and be subject to appropriate follow-up.
